# Portal Vein Thrombosis following Total Colectomy due to Colonic Inertia: A Case Report and Evaluation of Risk Factors

**DOI:** 10.1155/2021/8895206

**Published:** 2021-01-20

**Authors:** Mohammad Bagher Jahantab, Saadat Mehrabi, Vahid Salehi, Lotfolah Abedini, Mohammad Javad Yavari Barhaghtalab

**Affiliations:** Department of General Surgery, Shahid Beheshti Hospital, Yasuj University of Medical Sciences, Yasuj, Iran

## Abstract

The portal vein could be occluded by blood clots partially or completely causing portal vein thrombosis (PVT). The acute episode may be asymptomatic or manifested by abdominal pain, increasing body temperature, and unspecific dyspeptic symptoms. The main causes of PVT are categorized into local, acquired, and genetic thrombophilic factors. To our knowledge, this is the 2^nd^ recognized case of PVT  following colectomy for colonic inertia successfully treated with an effective anticoagulation therapy. The patient received unfractionated heparin as soon the diagnosis was implemented. The patient was a 34-year-old lady with chief complaint of severe abdominal pain, nausea, vomiting, and anorexia 10 days after the first hospital admission for subtotal colectomy due to colonic inertia. Spiral abdominal CT  scan with intravenous (IV) contrast showed thrombosis in main portal vein with its extension to right and left intrahepatic branches. Our case showed that we should keep in mind PVT in patients who present with upper gastrointestinal symptoms several days after a major surgery (open colectomy) as a risk factor and oral contraceptive pills (OCP) usage, postpregnancy, and immobility as other risk factors, that the protein C, S, and FVL deficiencies were secondary, and that the PVT can be managed by low molecular weight heparin plus oral warfarin therapy in the continue.

## 1. Background

Portal vein could be occluded by blood clot partially or completely causing portal vein thrombosis (PVT). Thrombosis could extend to intrahepatic branches and splenic or superior mesenteric veins (SMV). PVT is the result of a combination of local and systemic risk factors. Local risk factors accounts for about 20–25% of the cases and are categorized to cirrhotic and noncirrhotic patients. Noncirrhotic patients have malignant (solid tumors) or nonmalignant factors. Nonmalignant factors are intraabdominal infections such as cholecystitis, cholangitis, appendicitis, diverticulitis, pancreatitis, and inflammatory bowel diseases (IBD) and iatrogenic trauma to the portal system (splenectomy, Whipple, gastric bypass, hepatectomy, and liver transplantation), as well as liver and biliary system-related procedures such as cholecystectomy and hepatoportobiliary surgery. Systemic risk factors which account for about 50–60% of cases are acquired thrombophilia (antiphospholipid syndrome and paroxysmal nocturnal hemoglobinuria), inherited thrombophilia (mutations in factor V Leiden (FVL), factor II (prothrombin gene or G20210A), deficiency of protein C, S, plasminogen, and antithrombin III, myeloproliferative neoplasms (MPNs) (polycythemia vera, essential thrombocytemia, and primary myelofibrosis), autoimmune and rheumatologic disorders (autoimmune hepatitis, primary biliary cirrhosis, systemic lupus erythematosus, rheumatoid arthritis, Wegener's granulomatosis, mixed connective tissue disease, and Behcet syndrome), hyperhomocysteinemia, sickle cell disease, nephrotic syndrome, and thrombasthenia thrombocytosis. There are some other risk factors such as recent pregnancy, recent oral contraceptive use, male sex, and low platelet count [1–4]. It is important to know that in PVT pathogenesis, various underlying thrombophilic conditions may be existing [5].

Activated protein C (APC), protein S, and antithrombin are the most important natural anticoagulants [6]. Protein C or protein S deficiency frequency is estimated to be low in general population, but it is estimated to be 1.4–8.6% in venous thromboembolism (VTE) patients. An increased risk of thrombosis in a natural anticoagulant pathway is shown in protein C or S deficiencies. Half of the heterozygote individuals below the age of 50 years would confront a thrombotic event in their lives [7]. Prevalence of protein C and S deficiencies are shown to be 1–9% and 0–7% retrospectively in nonmalignant, noncirrhotic PVT  adult patients [8]. FVL has been shown to be in 3–9% of nonmalignant, noncirrhotic PVT adult patients and is the most frequent cause of inherited thrombophilia. Its pattern of inheritance is autosomal dominant [8, 9].

PVT  may occur rarely after colorectal procedures such as restorative proctocolectomy (RP) and ileal pouch-anal anastomosis (IPAA) and is associated to inflammatory conditions such as ulcerative colitis (UC) [10–13], and to a lower extent familial adenomatous polyposis and sigmoid cancer [14], and also colonic inertia [15].

Heterogeneity of the PVT clinical manifestations is due to the extent of the obstruction and the speed of its progress. The acute episode may be asymptomatic or manifested by abdominal pain, increasing body temperature, and nonspecific dyspeptic symptoms such as nausea and anorexia. Symptoms may be fewer or absent in partial PVT. Severe and colicky abdominal pain and diarrhea occur when the superior mesenteric vein is involved. With the progression of thrombosis, abdominal pain increases and radiation to the back may occur. In the next steps, ileus, intestinal ischemia, and infarction including hematochezia, ascites, metabolic acidosis, and renal or respiratory failure could be seen [4, 16]. The diagnosis is the absence of flow or the presence of a thrombus in the portal vein demonstrating in Doppler ultrasound, computerized tomography (CT), or magnetic resonance imaging (MRI) [4]. Improvement and increasing trend in the use of noninvasive radiological techniques in the diagnosis of abdominal pain has resulted in progressively more documented cases these days [16].

Colic inertia or slow transit constipation (STC) is a functional bowel pathology which may result in colonic incompetence to change stool consistency to move from the cecum to rectosigmoid for at least once every three days in existence of normal colon intraluminal diameter. If medical treatment fails in severe form of the disease, then several surgical treatment are indicated. These consist of subtotal colectomy with ileosigmoid or cecorectal anastomosis, segmental colectomy, and total abdominal colectomy with ileorectal anastomosis [17–19].

To our knowledge, this is the 2^nd^ recognized case of PVT following colectomy for colonic inertia who successfully treated with an effective anticoagulation therapy. In this study, we evaluated the risk factors for the formation of portal vein thrombosis following total colectomy due to colonic inertia.

## 2. Case Presentation

The patient was a 34-year-old lady who presented to emergency department with chief complaint of severe generalized colicky abdominal pain not radiating to any quadrant, nausea, not tolerating food ingestion, anorexia, and several episodes of nonprojectile, nonbilious, nonbloody vomiting per day, weakness, and letharginess about 10 days after the first hospital admission for subtotal colectomy due to colonic inertia. The patient had no loose stools and no mucus or blood in feces (melena or hematochezia), hematemesis, jaundice, and fever in days after discharge from the first hospitalization. There was no personal and familial history of venous thromboembolism; but she noted using oral contraceptive pills up to the day before first hospitalization for colectomy, but she denied using them after she was discharged from the 1^st^ hospitalization; she used oral cephalexin, pantoprazole, and acetaminophen after discharge from the 1^st^ admission. The patient had pregnancy about less than one year before the 1^st^ admission but had no previous abortions (G2P2L2A0).

On examination, the patient was oriented to time, place, and person with stable vital sign with a blood pressure of 100/70 mmHg and a pulse rate of 88/min, no icterus, edema, cyanosis or jaundice, and lymphadenopathy, but pale conjunctivae. There were clear breathing sounds and regular heart sounds without murmur. Patient had abdominal tenderness and rebound tenderness in periumbilical and right upper quadrant (RUQ) area, and there was no sign of hepatomegaly, splenomegaly, and wound infection in midline laparotomy site suture. Rectum was empty in rectal examination. Neurologic examination was normal. The patient's weight and height was 55 kg and 154 cm, respectively (body mass index (BMI) = 23.19).

During the 1^st^ hospitalization period, the patient received thromboembolic prophylaxis with heparin 5000 units subcutaneously twice a day, and also, the patient's both lower extremities were compressed by elastic bandages.

**Figure 1:**
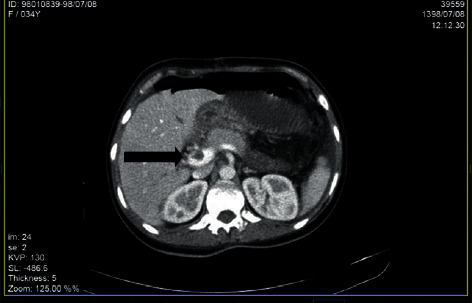
Thrombosis in main portal vein in spiral abdominal CT scan with IV contrast (black arrow).

**Figure 2:**
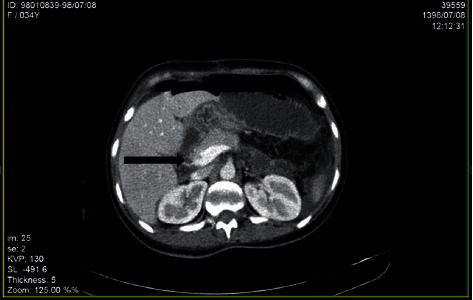
Thrombosis in main portal vein in spiral abdominal CT scan with IV contrast (black arrow).

**Figure 3:**
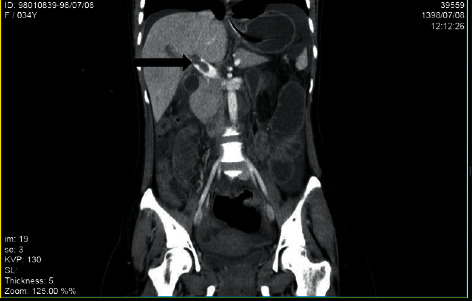
Thrombosis in main portal vein with its extension to right and left intrahepatic branches in spiral abdominal CT scan with IV contrast (black arrow).

Color Doppler ultrasonography showed no blood flow in right portal vein which suggested portal vein and portal confluence thrombosis about 25 mm and 13 mm in length retrospectively. Spiral abdominal CT scan with IV contrast showed thrombosis in main portal vein with its extension to right and left intrahepatic branches and dilatation of small bowel loops with the air-fluid level which suggested partial small bowel obstruction and free fluid in abdomen and pelvic spaces (Figures 1–3).

All laboratory studies were performed in hospital course, and results were as follows: serum blood sugar (BS) = 106 mg/dl, white blood cell (WBC) = 13600, hemoglobin (Hb) = 13.9 g/dl, platelet = 398000 U/L, erythrocyte sedimentation rate (ESR) = 27 mm/hr, C-reactive protein (CRP) = 49.1 mg/l, BUN = 7 mg/dl, creatinine = 0.7 mg/dl, Na^+^ = 139 mEq/L, K^+^ = 4.5 mEq/L, Mg = 1.74 mg/dl, serum amylase = 84 U/L, aspartate aminotransferase (AST) = 27 U/L, alanine aminotransferase (ALT) = 27 U/L, total protein = 5.2 gr/dl, albumin = 3.1 g/dl, total bilirubin = 0.34 mg/dl, direct bilirubin = 0.13 mg/dl, alkaline phosphatase = 128 U/L, homocysteine = 7.92 micmol/L, lactate dehydrogenase (LDH) = 329 U/L, anticardiolipin immunoglobulin M (IgM) = 1.56 GPLU/ml, anticardiolipin immunoglobulin G (IgG) = 2.55 IgG phospholipid units (GPLU)/ml, anti-B2-glycoprotein (IgM) = 9.48 U/ml, anti-B2-glycoprotein (IgG) = 9.88 U/ml, lupus anticoagulant (PTT-LA) = 38 second, Ca^++^ = 7.3 mg/dl (albumin = 3.1 g/dl), phos = 2.7 mg/dl, reticulocyte count = 2.3 corrected 0.95%, antithrombin III = 86%, FVL = 150 second, protein C = 61.57%, and protein S = 47.95%. Analysis of arterial blood gases (ABG) showed pH = 7.471, partial pressure of carbon dioxide (PCO_2_) = 31 mmHg, bicarbonate (HCO_3_) = 21 mmol/L, and base excess in the extracellular fluid compartment (BEecf) = −1.2 (interpretation = partly compensated respiratory alkalosis).

Heparin was started when results of cell blood count (CBC), activated partial thromboplastin time (aPTT), and prothrombin time (PT) were ready. The patient was administered therapeutic dosing of unfractionated heparin, 80 units/kg (5000 units) IV for loading dose and 1000 units IV continuous infusion for maintenance dose as soon as the diagnosis of PVT was made on the 1^st^ day of hospitalization. In order to monitor unfractionated heparin therapy, aPTT was checked every 6 hours to reach a therapeutic range of 1.5–2.5 times the control value (55–75 seconds). To exclude heparin-induced thrombocytopenia and also finding out if Hb drop occurs, CBC was performed every other day. When aPTT reached the therapeutic level in the 2^nd^ day after heparin administration, warfarin 5 mg daily was started, and afterward, the patient was monitored by aPTT and international normalized ratio (INR). On the 6^th^ day and 4^th^ day after heparin and warfarin administration respectively, INR became more than 2 (therapeutic level), so heparin continued for two other days afterward; then, it was discontinued, and the patient was discharged home in stable vital sign and good condition on the 9^th^ day of hospital admission with warfarin 5 mg daily. In hospital course, there was no adverse or anticipated event.

The patient had follow-up visits on the 7^th^day and 30^th^ day after discharge from hospital, and there were no complications, and the patient came back to her daily routine activities successfully.

## 3. Discussion and Conclusions

Portal vein thrombosis is a rare disease with severe complications and would cause variceal bleeding and intestinal ischemia if left untreated. The main causes of PVT are categorized in local, acquired, and genetic thrombophilic factors [20]. In our patient, there were some possible risk factors for developing PVT, a major surgery as a local risk factor (colectomy in this patient), and acquired risk factors as OCP usage, postpregnancy, and immobility, but there were no genetic thrombophilic factors found in this patient.

The risk of PVT  after colectomy may be due to ligation of the ileocolic vessels, mobilization of the small intestinal mesentery from the posterior abdominal wall, tension on the mesenteric vessels in the time of bowel loop anastomosis, and direct trauma to the port (it is doubtful because portal vein is located behind the pancreas) [12, 21]. Inflammatory processes such as IBD and diverticulitis are shown to induce PVT after laparoscopic colectomy [22, 23]; however, our patient had no IBD associated with postoperative PVT.

Other risk factors in developing thrombosis include pregnancy or oral contraceptive usage. OCP is a combination of an estrogen (ethinylestradiol) and a progestogen (progestin). Estrogen may cause an increase in procoagulant factors such as prothrombin, VII, X, XII, and XIII, resistance to activated protein C, and fibrinolysis. Progestin may increase the estrogen effect on procoagulant, anticoagulant, and fibrinolytic pathways [24].

In a study performed by Fisher et al. on 29 adult PVT patients, it was found that 18 patients (62%) had deficiencies in one or more of the natural anticoagulant proteins (proteins C, S, or antithrombin), 8 cases (28%) had combined C and S protein deficiency, and 6 cases (21%) had combined deficiency of all three proteins. As far as our knowledge, this report is the first of its own which presents combination of these two protein deficiencies plus FVL deficiency. They proposed that these deficiencies are acquired dominantly, and that is a result of PVT, but a minority of cases have a genetic background for anticoagulant protein deficiency, so a careful evaluation and also consultation of family members (preferably both parents and alternatively siblings) should be conducted [25]. In our case, there was limitation in investigating the patient's family members (her parents and sibling).

Two probable mechanisms were proposed for reduction of these natural anticoagulant proteins in patients who developed PVT: (1) reduction in liver blood flow would cause hepatic atrophy and reduce hepatic protein synthesis (coagulation factors more than albumin). (2) Reduction in coagulation proteins concentration due to increased clearance after shunting of blood from the liver [26].

Nevertheless, there is a related predisposing factor; patients presenting with PVT should be investigated for thrombophilia through checking of CBC, ESR, PT, aPTT, thrombin time (TT), peripheral blood smear (PBS), antithrombin III (AT III), fibrinogen level, proteins C and S, and FLV [20]. In our patient, as we checked proteins C, S, and FVL during the disease process at the hospital course, we could say that the deficiencies in these factors are acquired and could be secondary to the manipulation of the abdomen after the operation.

In conclusion, our case showed that we should keep in mind PVT in patients who present with upper gastrointestinal symptoms several days after a major surgery (open colectomy) as a risk factor; OCP usage, postpregnancy, and immobility as other risk factors, that the proteins C, S, and FVL deficiencies were acquired and secondary to the abdominal operation, and that the PVT can be managed by low molecular weight heparin plus oral warfarin therapy in the continue.

## Data Availability

The data used to support the findings of this study are available from the corresponding author upon request.

## Abbreviations

FVL: Factor V Leiden
IV: Intravenous
OCP: Oral contraceptive pill
PVT: Portal vein thrombosis
SMV: Superior mesenteric veins
IBD: Inflammatory bowel diseases
MPNs: Myeloproliferative neoplasms
APC: Activated protein C
VTE: Venous thromboembolism
RP: Restorative proctocolectomy
IPAA: Ileal pouch-anal anastomosis
UC: Ulcerative colitis
CT: Computerized tomography
MRI: Magnetic resonance imaging
STC: Slow transit constipation
RUQ: Right upper quadrant
BS: Blood sugar
Hb: Hemoglobin
LDH: Lactate dehydrogenase
ABG: Arterial blood gases
ESR: Erythrocyte sedimentation rate
CRP: C-reactive protein
CBC: Cell blood count
PT: Prothrombin time
aPTT: Activated partial thromboplastin time
PBS: Peripheral blood smear
TT: Thrombin time
AT III: Antithrombin III.
